# Paroxetine suppresses reactive microglia-mediated but not lipopolysaccharide-induced inflammatory responses in primary astrocytes

**DOI:** 10.1186/s12974-020-1712-0

**Published:** 2020-02-05

**Authors:** Xiong Zhang, Lan-Bing Zhu, Jia-Hui He, Hong-Qiu Zhang, Shu-Ya Ji, Chao-Nan Zhang, Na-Na Hou, Chen-Ping Huang, Jian-Hong Zhu

**Affiliations:** 1 Department of Geriatrics and Neurology, the Second Affiliated Hospital and Yuying Children’s Hospital, Wenzhou Medical University, Wenzhou, Zhejiang, 325027 China; 2 Department of Preventive Medicine, Wenzhou Medical University, Wenzhou, Zhejiang, 325035 China

**Keywords:** Paroxetine, Astrocytes, Microglia, Neuroinflammation, Parkinson’s disease

## Abstract

**Background:**

Astrocytes are the most abundant glial cells in a brain that mediate inflammatory responses and provide trophic support for neurons. We have previously disclosed that paroxetine, a common selective serotonin reuptake inhibitor, ameliorates LPS-induced microglia activation. However, it remains elusive for the role of paroxetine in astrocytic responses.

**Methods:**

Isolated primary astrocytes were pretreated with paroxetine and stimulated with different stimuli, lipopolysaccharide (LPS) or microglia conditioned medium pre-activated with LPS (M/Lps). Inflammatory and neurotrophic responses, underlying mechanisms and the impact on neuronal survival were assessed.

**Results:**

Paroxetine had no impact on LPS-stimulated iNOS, TNF-α, and IL-1β expression, but inhibited M/Lps-induced TNF-α and IL-1β expression in primary astrocytes. Paroxetine suppressed M/Lps- but not LPS-induced activation of NF-κB and had no impact on the activation of MAPKs and STAT3. Incubation with the resulted astrocyte conditioned media caused no change in the viability of SH-SY5Y cells. BDNF and MANF mRNA expressions were upregulated by M/Lps and paroxetine, respectively. However, M/Lps- or LPS-induced extracellular releases of NO, TNF-α, and/or BDNF in astrocytes were in minor amount compared to those by microglia.

**Conclusions:**

Paroxetine ameliorates the reactive microglia-mediated inflammatory responses in astrocytes partially via inhibition of the NF-κB pathway but has no impact on LPS-stimulated astrocyte activation. While the effects of paroxetine on secondary astrocytic responses are not robust compared to its effect on the innate immune responses of microglia, the results together may implicate a therapeutic potential of paroxetine against neuroinflammation-associated neurological disorders such as Parkinson’s disease.

## Background

Parkinson’s disease (PD) is a common neurodegenerative disease characterized by the selective death of dopaminergic neurons in the substantia nigra. Although its exact etiology remains elusive, accumulating evidence has suggested that neuroinflammation plays a key role in the pathogenesis of PD [1–3]. In the central nervous system, innate immune responses are collectively mediated by microglia and astrocytes while the former dominates the way to neuroinflammation [4, 5]. Indeed, reactive microglia and astrocytes are both found in the nigrostriatal bundle of PD patients or PD animal models [6–9]. A number of PD-associated gene products such as α-synuclein, PINK1, and DJ-1 have been implicated in astrocyte dysfunction. Exposure to mutant α-synuclein and deficiency in PINK1 lead to activation of both microglia and astrocytes and generation of a large amount of neuroinflammatory factors [10–12]. PINK1 deficiency also inhibits the differentiation of neural stem cells into astrocytes [13] and causes proliferation defect in astrocytes which may result in a delay in the wound healing process [14]. DJ-1 is abundantly expressed in reactive astrocytes of PD patients [15]. Its deficiency impairs glutamate uptake into astrocytes [16] and leads to increased susceptibility to inflammatory signaling [17]. Different from microglia, reactive astrocytes depending on the context may release pro-inflammatory factors such as tumor necrosis factor α (TNF-α) and interleukin-1β (IL-1β), or provide trophic support for neurons by releasing neurotrophic factors such as brain-derived neurotrophic factor (BDNF) and mesencephalic astrocyte-derived neurotrophic factor (MANF) [4, 5, 18]. The interaction between microglia and astrocytes leads to collective outcomes for neurons. For instance, astrocytes were reported to enhance the inflammatory responses of activated microglia, resulting in more dopaminergic neuron death [19].

Studies have suggested the negative immunoregulatory effects of antidepressant drugs [20–23]. Amongst, paroxetine is a common selective serotonin reuptake inhibitor and is used for disorders such as major depressive disorder, generalized anxiety disorder, and obsessive-compulsive disorder, and also with fewer side effects than the first generation antidepressants tricyclic antidepressants [24, 25]. Depression is the most common psychiatric disturbance reported in PD patients. Paroxetine is thus also used for relieving depressive symptomatology and regulating behavioral control in PD patients and is generally well tolerated [26, 27]. Paroxetine is reported to protect against dopaminergic neuronal loss in an MPTP-induced mouse model of PD, potentially through its inflammatory alleviation in the substantia nigra [28]. Lipopolysaccharide (LPS) is an endotoxin often used for modeling PD in the context of neuroinflammation both in vivo [29, 30] and in vitro [31–35], particularly when the neuroinflammation aspect of mechanisms is focused. By applying LPS, we have previously disclosed that paroxetine ameliorates the microglia activation through regulation of MAPK signaling [36]. However, it remains elusive for the role of paroxetine in astrocytic responses. Thus, in this study, we aimed to understand the impact of paroxetine on astrocyte activation induced by LPS and reactive microglia using a conditioned medium culture system.

## Methods

### Cell culture and primary astrocyte isolation

Dulbecco’s modified eagle medium (DMEM; C11995500BT) and DMEM/F-12 (C11330500BT) were purchased from Gibco (Grand Island, NY, USA). Fetal bovine serum (FBS; VS500T, Australia origin) was from Ausbian (Shanghai, China). BV2 microglia cells (provided by Dr. Zhu CQ, Fudan University) and SH-SY5Y cells (Cell Bank of Chinese Academy of Sciences, Shanghai, China) were grown in DMEM supplemented with 10% FBS and penicillin/streptomycin (100 U/mL and 100 μg/mL, respectively; P1400, Solarbio, Beijing, China). Cells were cultured at 37 °C in a humidified atmosphere of 5% CO_2_.

**Fig. 1:**
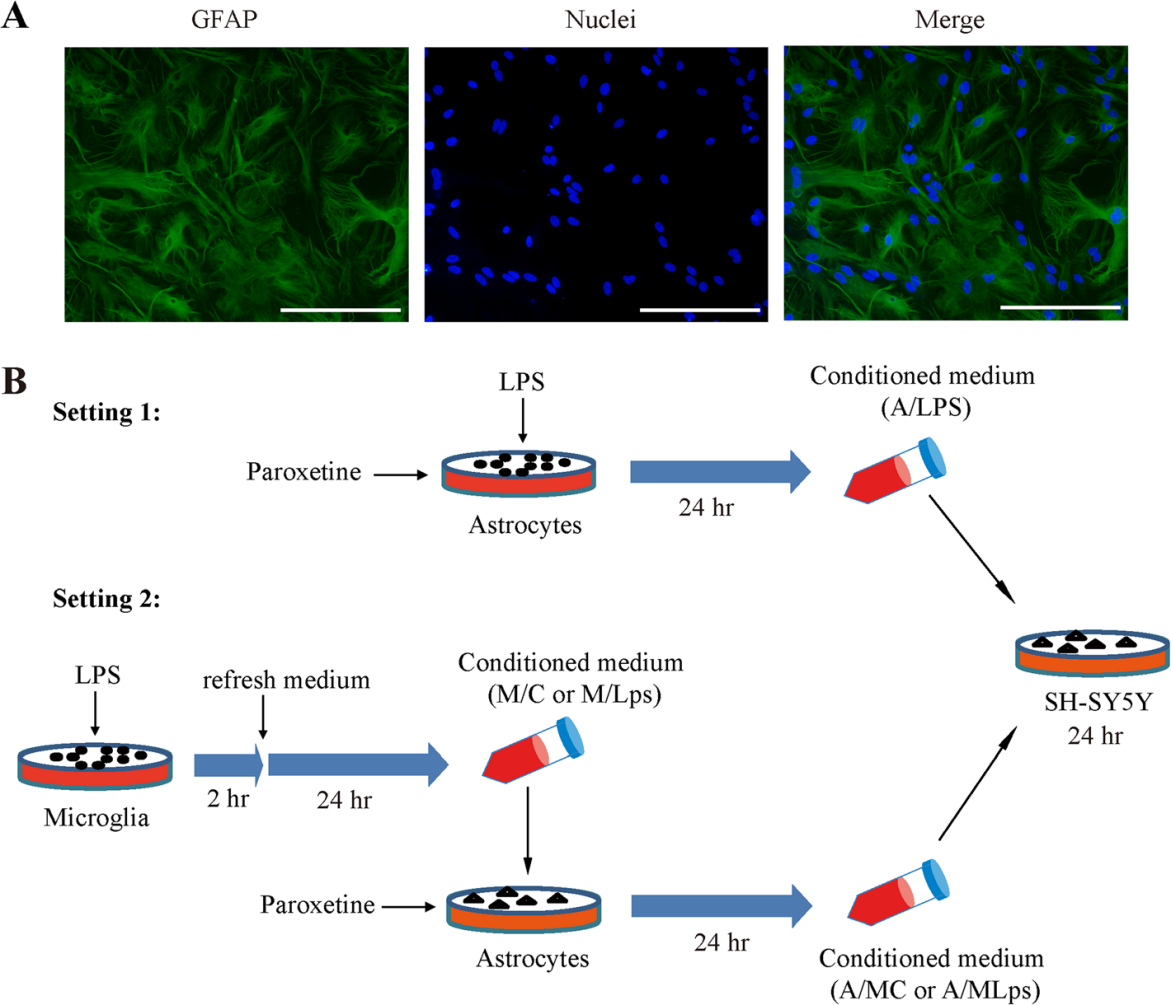
Isolation of primary astrocytes and experimental design. **a** Representative images of isolated primary astrocytes. Green, GFAP; blue, nuclei; bar size, 100 μm. **b** Illustration of experimental design and conditioned media. A/Lps, conditioned medium of astrocytes cultured with LPS; A/MC, conditioned medium of astrocytes cultured with M/C; A/MLps, conditioned medium of astrocytes cultured with M/Lps; GFAP, glial fibrillary acidic protein; LPS, lipopolysaccharide; M/C, microglia conditioned medium without LPS stimulation; M/Lps, microglia conditioned medium pre-activated with LPS

Primary astrocytes were prepared as previously described with slight modification [37, 38]. In brief, cerebral cortices were dissected from brains of new-born pups of the Institute of Cancer Research (ICR) mice (postnatal 1–2 days; purchased from the Experimental Animal Center of Wenzhou Medical University) in cold Hank’s buffered saline. The animals used in this study were treated in accordance with protocols approved by the Institutional Animal Care and Use Committee of Wenzhou Medical University. Cortices were gently triturated and digested with 0.25% Trypsin-EDTA solution (25,200,056, Gibco, Grand Island, NY, USA) for 15 min at 37 °C. Thereafter, an equal volume of culture medium, that is, DMEM/F-12 supplemented with 10% FBS and penicillin/streptomycin, was added and centrifuged at 200 g for 5 min. The pellets were gently resuspended in culture medium and filtered through a 100-μm-pore mesh. The cells were then seeded in 75 cm^2^ flasks coated with poly-L-lysine (1 mg/mL) and cultured at 37 °C with medium changed every 4 days. When cells reached a confluency of 90–95%, the flasks were placed on an orbital shaker at 250 rpm and 37 °C for 16 h. The remaining attached cells were then collected and re-cultured. The purity of astrocytes was evaluated by immunostaining of glial fibrillary acidic protein (GFAP). GFAP positive cells were counted in 3-random fields each time. Cultures were used for subsequent experiments when GFAP positivity was over 95% (Fig. 1a).

### Immunofluorescence

Cells were fixed in 4% paraformaldehyde for 30 min, followed by permeabilization in 0.2% Triton X-100 for 15 min and then blocked with 5% bovine serum albumin (Beyotime, Shanghai, China) for 1 h at room temperature. The washing buffer is phosphate-buffered saline. Primary antibodies against GFAP and p65 were from Millipore (MAB360, Billerica, MA, USA) and Cell Signaling (8242S, Boston, MA, USA), respectively. Alexa Fluor 488-conjugated anti-mouse (A11001) and 555-conjugated anti-rabbit (A21428) IgG antibodies and Hoechst 33258 (H3569) were from Thermo Fisher (Rockford, IL, USA).

### Treatments and preparation of conditioned media

Microglia conditioned media were prepared as previously [19]. BV2 cells were seeded at a density of 2–3 × 10^6^ in a 10-cm culture plate and cultured for 24 h followed by serum starvation overnight. Cells were then treated with or without LPS (100 ng/mL; L4391, Sigma, St. Louis, MO, USA) for 2 h, and then refreshed with serum-free medium with a continued incubation for 24 h. Following centrifugation at 20,000 g for 5 min, the media were collected as microglia conditioned medium without LPS stimulation (M/C) or microglia conditioned medium pre-activated with LPS (M/Lps), and stored at − 80 °C till use (Fig. 1b).

Primary astrocytes of passage 3–5 were seeded at a density of 2–3 × 10^6^ per well in a 6-well plate and cultured for 24 h. Following starvation overnight, cells were pretreated with paroxetine (P9623, Sigma, St. Louis, MO, USA) at indicated concentrations for 30 min, followed by treatments of LPS (100 ng/mL) or the above M/C and M/Lps for the indicated time. The resulted media of astrocytes treated with M/C, M/Lps or LPS for 24 h were centrifuged at 20,000 g for 5 min and designated as A/MC (conditioned medium of astrocytes cultured with M/C), A/MLps (conditioned medium of astrocytes cultured with M/Lps), and A/Lps (conditioned medium of astrocytes cultured with LPS), respectively (Fig. 1b). Media were stored at − 80 °C till use.

### Cell viability measurement

Cell viability was determined by the tetrazolium salt 3-[4,5-dimethylthiazol-2-yl] -2,5-diphenyltetrazolium bromide (MTT; C0009, Beyotime, Shanghai, China) assay [39]. Primary astrocytes or SH-SY5Y cells were seeded in triplicates in 96-well plates at 5 × 10^4^ and 1 × 10^4^ per well, respectively, and cultured for 24 h. Astrocytes were treated with paroxetine at different concentrations for 24 h following overnight starvation. SH-SY5Y cells were incubated with A/MC, A/MLps, A/Lps, or LPS directly (100 ng/mL) for 24 h. Thereafter, MTT (5 mg/mL prepared in phosphate-buffered saline) was added into each well and incubated at 37 °C for 4 h. The resulting formazan crystals were dissolved in dimethyl sulfoxide. Optical density was measured at 490 nm.

### RNA isolation and quantitative PCR

Total RNA was isolated using TRIzol Reagent (15596018, Invitrogen, Carlsbad, CA, USA), and reverse-transcribed to cDNA using the PrimeScript RT kit (RR037A, TaKaRa, Dalian, China). Quantitative PCR was performed using SYBR Green Supermix (1725260, Bio-Rad, Hercules, CA, USA) following the manufacturer’s instruction. Primers were as follows: TNF-α, 5′-CGT CAG CCG ATT TGC TAT CT-3′ and 5′-CGG ACT CCG CAA AGT CTA AG-3′; IL-1β, 5′-GAA ATG CCA CCT TTT GAC AGT G-3′ and 5′-TGG ATG CTC TCA TCA GGA CAG-3′; BDNF, 5′-TCA TAC TTC GGT TGC ATG AAG G-3′ and 5′-AGA CCT CTC GAA CCT GCC C-3′; MANF, 5′-TCT GGG ACG ATT TTA CCA GGA-3′ and 5′-CTT GCT TCA CGG CAA AAC TTT-3′; β-actin, 5′-AGC CAT GTA CGT AGC CAT CC-3′ and 5′-CTC TCA GCT GTG GTG GTG AA-3′.

### Determination of TNF-α and BDNF production

Medium TNF-α and BDNF were measured using ELISA kits respectively from R&D Systems (VAL609, Minneapolis, MN, USA) and Westang (F10200, Shanghai, China) according to the manufacturers’ instructions. Absorbance was read at 450 nm. The concentration of each sample was calculated from the standard curve prepared using the included standards.

### NO production assay

Medium nitrite was measured as an indicator of NO production [40]. In brief, 50 μl of supernatant was mixed with an equal volume of Griess reagent I, followed by the addition of 50 μl of Griess reagent II (S0021, Beyotime, Shanghai, China) at room temperature. Absorbance was immediately measured at 540 nm. The concentration of each sample was calculated from a standard curve generated using sodium nitrite.

### Western blot analysis

Cells were lysed in sample buffer containing 60 mM Tri-HCl, pH 6.8, 2% SDS and 5% glycerol, and boiled for 5 min. Total protein concentration was measured using a BCA kit (P0010, Beyotime, Shanghai, China). An equal amount of protein from each sample was loaded and analyzed by western blot as previously described [41]. Pyrrolidine dithiocarbamic acid (PDTC; P8765) was purchased from Sigma (St. Louis, MO, USA). Primary antibodies against p-p65 (3031), p65 (3034), p-JNK1/2 (9251), JNK1/2 (9252), p-p38 (9211), p38 (9212), p-ERK1/2 (9101), ERK1/2 (9102), p-STAT3 (9145), STAT3 (12640), iNOS (2977) and β-actin (4970), anti-rabbit (7074) and anti-mouse (7076) secondary antibodies, and LumiGLO® Reagent and Peroxide chemiluminescence detection kit (7003) were all purchased from Cell Signaling (Boston, MA, USA).

### Statistical analysis

Data were analyzed by one-way analysis of variance (ANOVA) followed by Tukey’s post hoc test for multiple comparisons and two-way ANOVA for factorial design experiments using the SPSS 23.0 statistics software. Values were expressed as mean ± SE from at least three independent experiments. The *p* < 0.05 was considered statistically significant.

## Results

### Effect of paroxetine on LPS-induced inflammatory responses in primary astrocytes

**Fig. 2:**
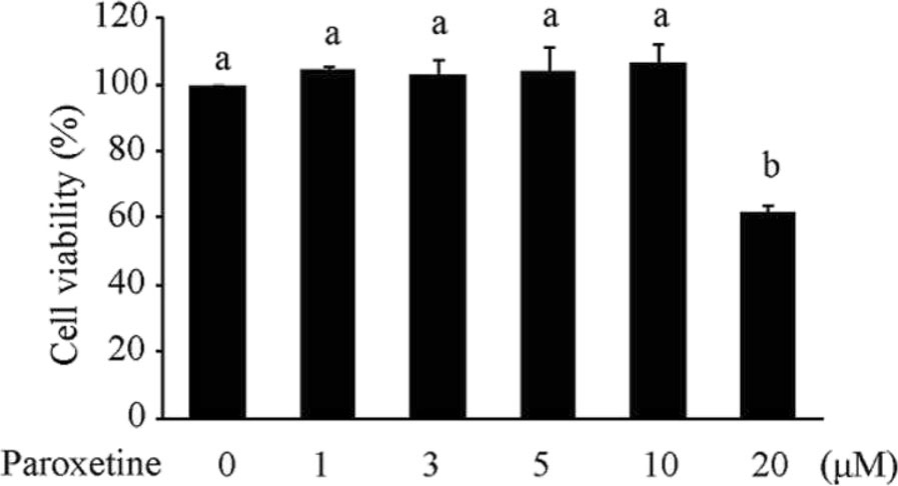
Impact of paroxetine on the viability of primary astrocytes. Cells were treated with paroxetine at different concentrations for 24 h. Cell viability was expressed as percentage of the control (0 μM), which was set as 100%. Values are means ± SE, *n* = 3. Statistical comparisons were performed using one-way ANOVA, followed by Tukey’s post hoc test. Different letters indicate *p* < 0.05

To understand the potential toxicity of paroxetine on primary astrocytes, we treated cells with paroxetine at 0, 1, 3, 5, 10, and 20 μM for 24 h. Results showed that paroxetine at 20 μM, but not at the other doses, induced significant cell death [Fig. 2; *F*_(5,12)_ = 15.38, *p* < 0.001; post hoc, *p* = 0.001 (0 μM vs 20 μM)].

**Fig. 3:**
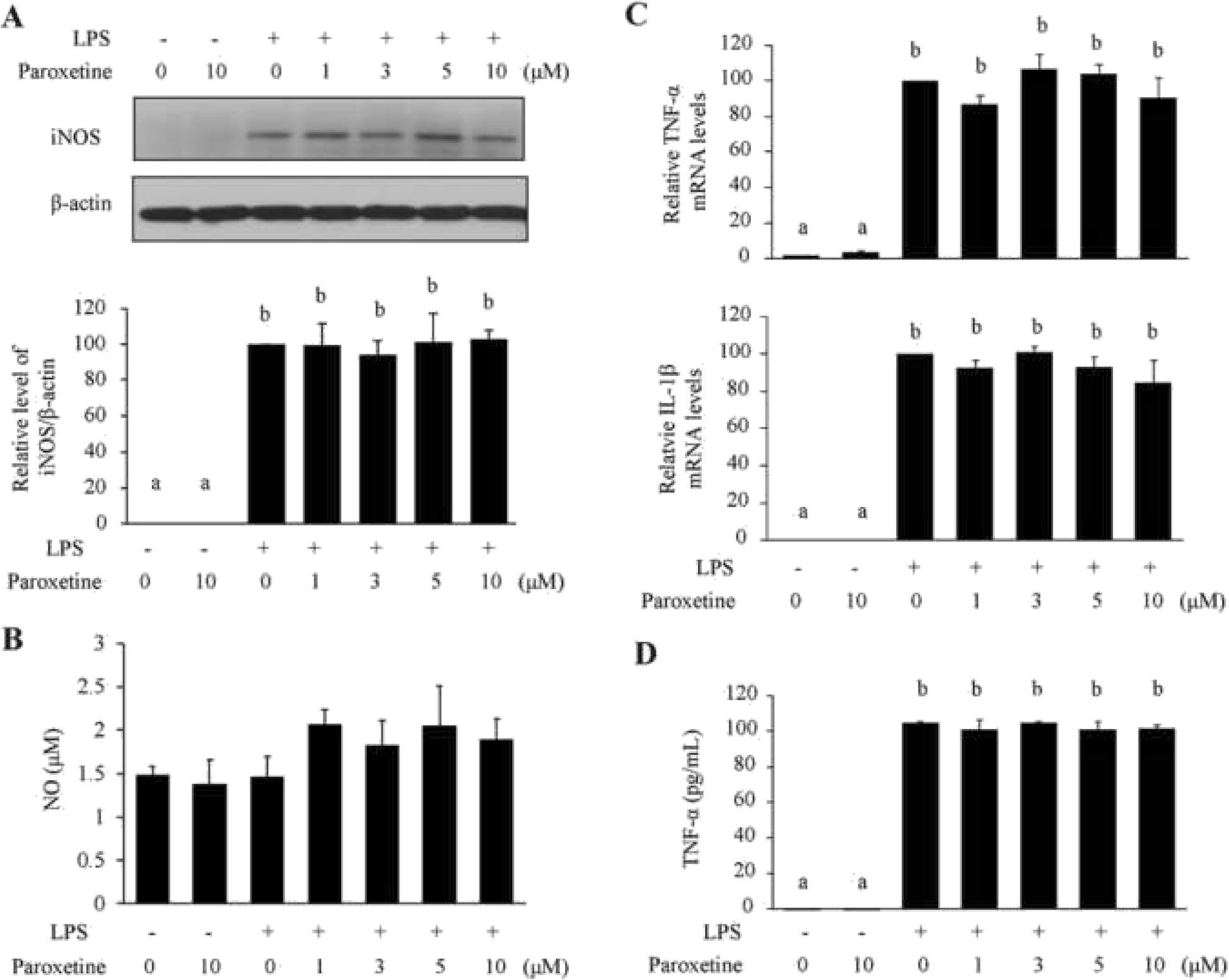
Effect of paroxetine on LPS-induced inflammatory responses in primary astrocytes. Cells were pretreated with paroxetine at different concentrations for 30 min followed by stimulation with LPS at 100 ng/mL for 24 h. **a** Western blot analysis of iNOS expression. Protein levels were quantified and normalized to their respective β-actin levels. Values were expressed relative to the one treated with LPS alone, which was set as 100. **b** NO production indicated by nitrite levels in culture media. **c** Quantitative PCR analyses of TNF-α and IL-1β expression. The mRNA levels were expressed relative to the one treated with LPS alone, which was set as 100. **d** Concentrations of TNF-α in culture media. Values are means ± SE, *n* = 3 for **a**, **b**, and **d**, *n* = 4 for **c**. Statistical comparisons were performed using one-way ANOVA, followed by Tukey’s post hoc test. Different letters indicate *p* < 0.05. IL-1β, interleukin 1β; iNOS, inducible nitric oxide synthase; LPS, lipopolysaccharide; NO, nitric oxide; TNF-α, tumor necrosis factor α

LPS stimulated light iNOS expression in isolated primary astrocytes [Fig. 3a; *F*_(6,14)_ = 31.82, *p* < 0.001; full blots listed in Additional file 1: Figure S1], as the induction is much weaker than that in microglia following the same treatment [36]. As a consequence, no significant elevation in NO levels was detected in astrocytes upon LPS treatment (Fig. 3b). LPS induced a significant increase in TNF-α and IL-1β mRNA expression [Fig. 3c; TNF-α: *F*_(6,21)_ = 56.12, *p* < 0.001; IL-1β: *F*_(6,21)_ = 67.68, *p* < 0.001] and extracellular release of TNF-α [Fig. 3d; *F*_(6,14)_ = 225.56, *p* < 0.001]. However, paroxetine pretreatments at different concentrations did not lead to apparent changes in iNOS expression and NO production (Fig. 3a, b), neither in TNF-α and IL-1β mRNA expression and TNF-α production (Fig. 3c, d).

### Effect of paroxetine on M/Lps-induced inflammatory responses in primary astrocytes

**Fig. 4:**
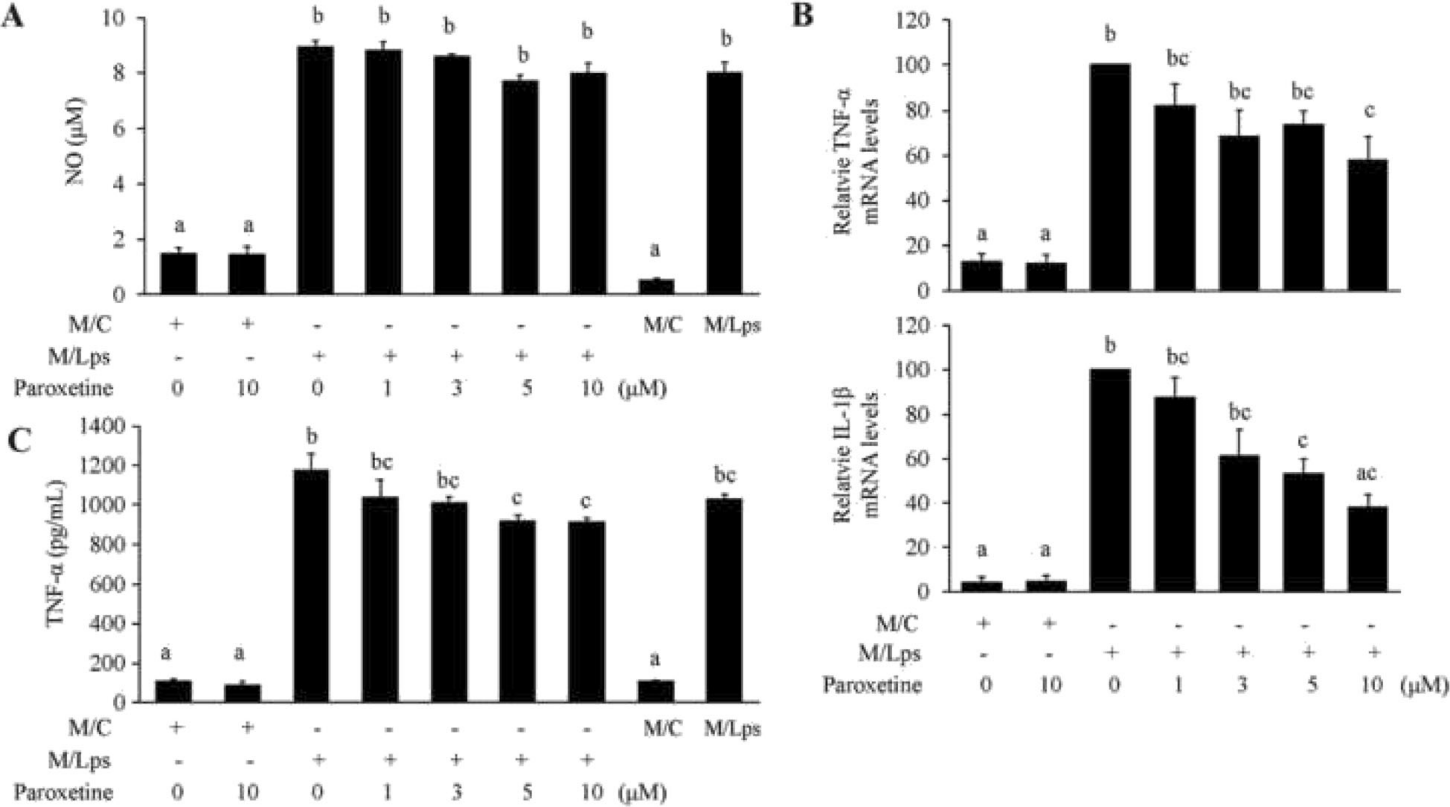
Paroxetine suppresses M/Lps-induced inflammatory responses in primary astrocytes. Cells were pretreated with paroxetine at different concentrations for 30 min followed by incubation with microglia conditioned media for 24 h. **a** NO production indicated by nitrite levels in culture media. **b** Quantitative PCR analyses of TNF-α and IL-1β expression. The mRNA levels were expressed relative to the one incubated with M/Lps alone, which was set as 100. **c** Concentrations of TNF-α in culture media. Values are means ± SE, *n* = 3 for **a**, *n* = 4 for **b**, **c**. Statistical comparisons were performed using one-way ANOVA, followed by Tukey’s post hoc test. Different letters indicate *p* < 0.05. IL-1β, interleukin 1β; M/C, microglia conditioned medium without LPS stimulation; M/Lps, microglia conditioned medium pre-activated with LPS; NO, nitric oxide; TNF-α, tumor necrosis factor α

The iNOS expression upon M/Lps incubation was barely detectable (data not shown). To determine whether there was an increase in the production of cytokines derived from astrocytes, the NO, and TNF-α carried from M/C or M/Lps per se (without treating astrocytes) were measured being their baseline levels (Fig. 4a, c; the right last two columns). There was no significant production of NO by astrocytes treated with M/Lps compared to its baseline level (Fig. 4a). M/Lps induced a significant increase in TNF-α and IL-1β mRNA expression in primary astrocytes. The induction was suppressed by pretreatments of paroxetine, in particular at doses of 5 and 10 μM [Fig. 4b; TNF-α: *F*_(6,21)_ = 14.77, *p* < 0.001; post hoc, *p* = 0.037 (M/Lps vs M/Lps + paroxetine 10 μM; down by 42.2%); IL-1β: *F*_(6,21)_ = 15.88, *p* < 0.001; post hoc, *p* = 0.027 (M/Lps vs M/Lps + paroxetine 5 μM; down by 46.7%), *p* = 0.002 (M/Lps vs M/Lps + paroxetine 10 μM; down by 61.8%)]. Although not significant, a slight increase appeared in the production of TNF-α in media of astrocytes incubated with M/Lps compared to its baseline level (the third vs the ninth). This increase was blunted by pretreatments of paroxetine at doses of 5 and 10 μM [Fig. 4c; *F*_(8,27)_ = 99.06, *p* < 0.001; post hoc, *p* = 0.013 (M/Lps vs M/Lps + paroxetine 5 μM; down by 21.9%), *p* = 0.011 (M/Lps vs M/Lps + paroxetine 10 μM; down by 22.3%)].

### Paroxetine inhibits M/Lps-induced inflammatory responses in primary astrocytes partially through NF-κB pathway

**Fig. 5:**
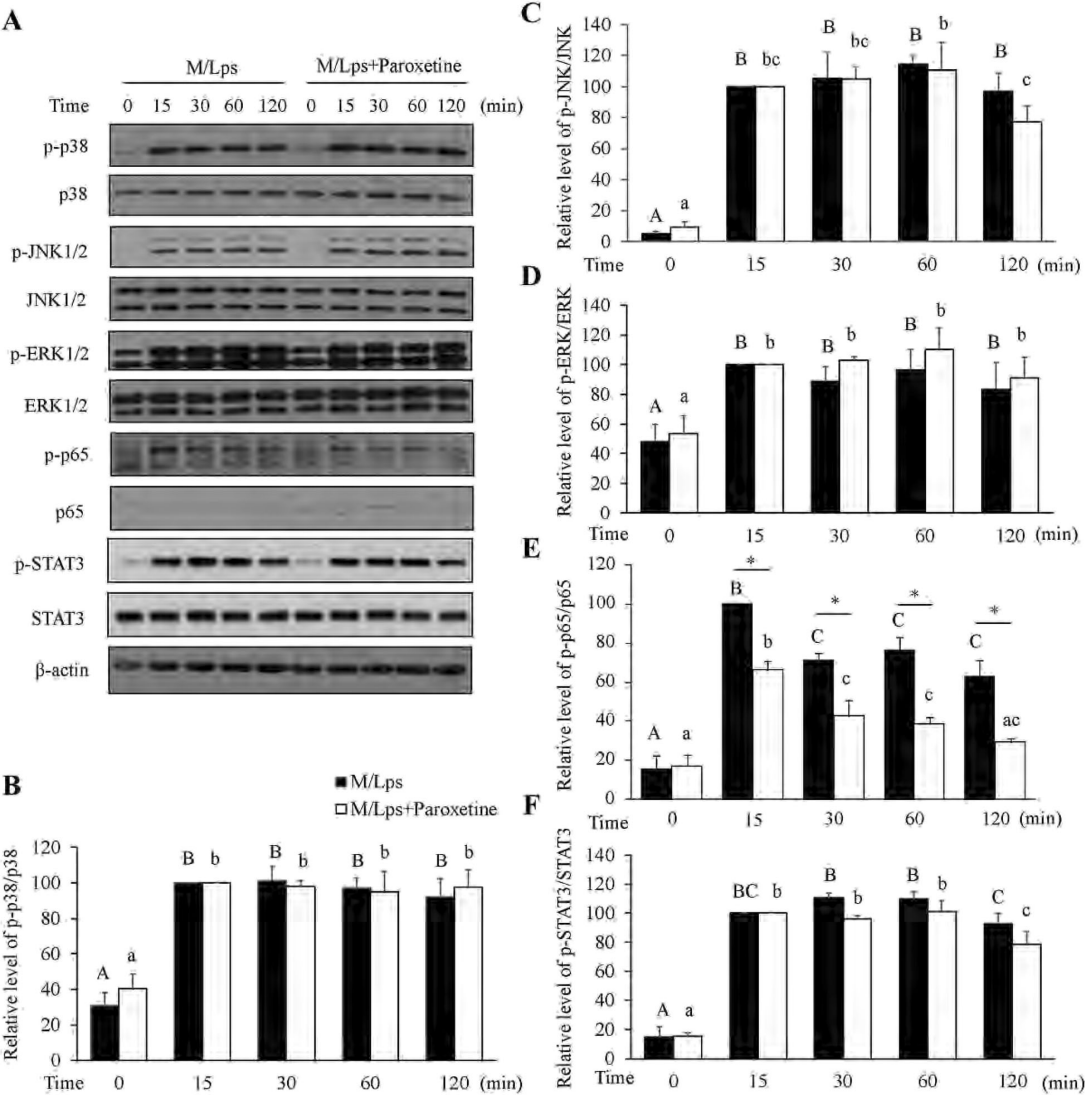
Effect of paroxetine on M/Lps-induced signaling activation in primary astrocytes. Cells were pretreated with or without 10 μM of paroxetine for 30 min followed by stimulation with M/Lps for 0–120 min. **a** Western blot analyses of p38, JNK1/2, ERK1/2, p65/NF-κB, and STAT3 activation. **b–f** Levels of phosphorylation forms were quantified and normalized to their respective total levels. Values were expressed relative to the one stimulated with M/Lps alone for 15 min, which was set as 100. Data are means ± SE, *n* = 4. Statistical comparisons were performed using two-way ANOVA. Different letters and * indicate *p* < 0.05. M/Lps, microglia conditioned medium pre-activated with LPS

**Fig. 6:**
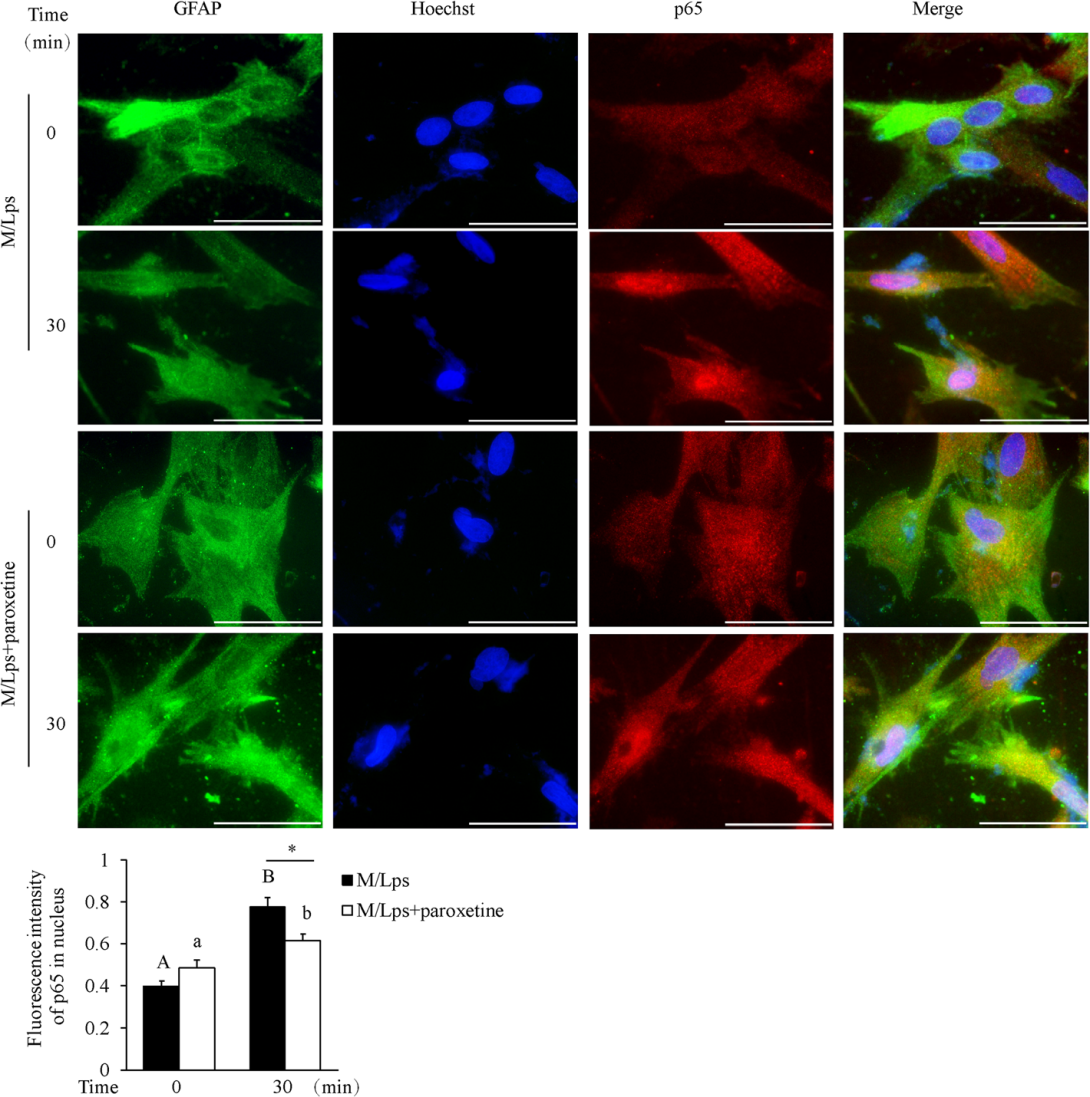
Effect of paroxetine on M/Lps-induced p65/NF-κB nuclear translocation in astrocytes. Cells were pretreated with or without 10 μM of paroxetine for 30 min followed by stimulation with M/Lps for 30 min. **a** Representative images of p65 immunostaining. Green, GFAP; red, p65; blue, nuclei; bar size, 200 μm. **b** Fluorescence intensity of nuclear p65 was determined by quantifying 15 cells in multiple random fields, followed by normalization to their respective nuclei staining. Data are expressed as means ± SE from three independent experiments. Statistical comparisons were performed using two-way ANOVA. Different letters and * indicate *p* < 0.05. GFAP, glial fibrillary acidic protein; M/Lps, microglia conditioned medium pre-activated with LPS

**Fig. 7:**
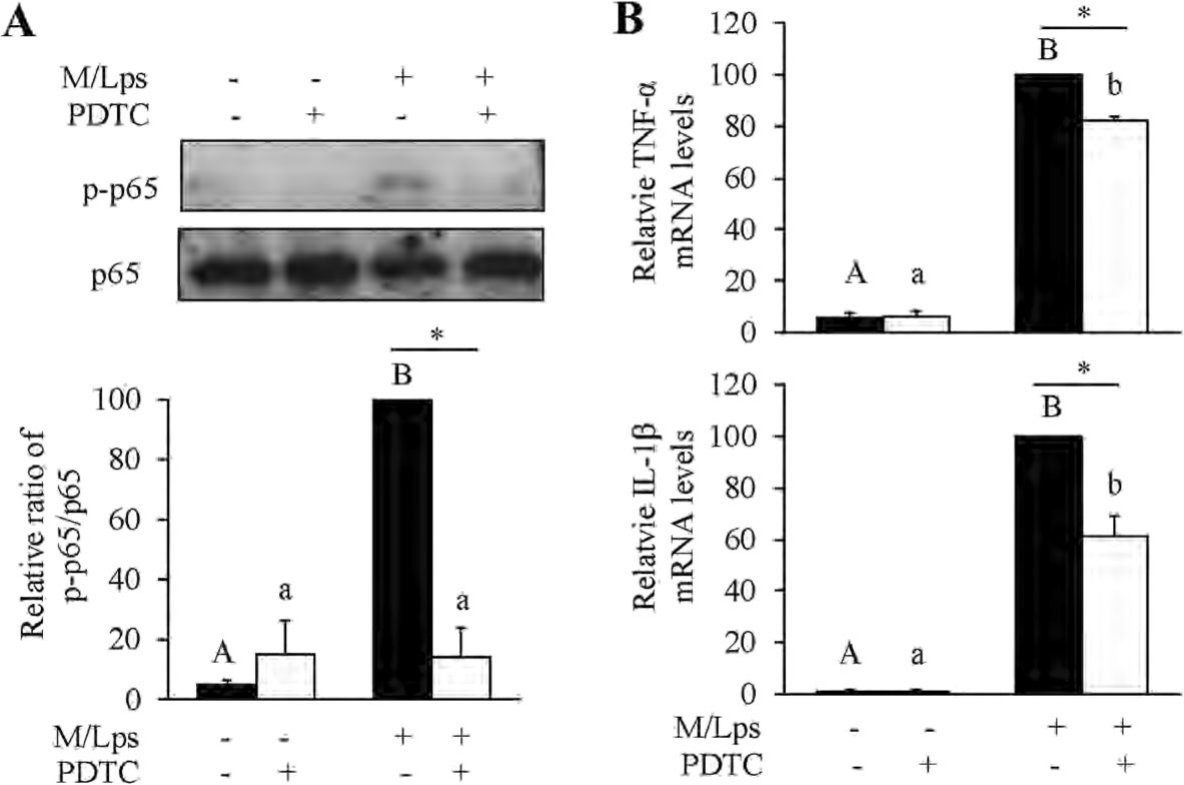
Inhibition of p65/NF-κB signaling on M/Lps-stimulated inflammatory responses in astrocytes. **a** Effect of PDTC on p65 activation. Cells were pretreated with 20 μM of PDTC for 2 h prior to stimulation with M/Lps for 30 min. Levels of p-p65 were quantified and normalized to total p65 levels. **b** Effect of PDTC on M/Lps-induced mRNA expression of TNF-α and IL-1β. Cells were pretreated with 20 μM of PDTC for 2 h prior to stimulation with M/Lps for 24 h. Values were expressed relative to the one stimulated with M/Lps alone, which was set as 100. Data are means ± SE, *n* = 4. Statistical comparisons were performed using two-way ANOVA. Different letters and * indicate *p* < 0.05. IL-1β, interleukin 1β; M/C, microglia conditioned medium without LPS stimulation; M/Lps, microglia conditioned medium pre-activated with LPS; PDTC, pyrrolidine dithiocarbamic acid; TNF-α, tumor necrosis factor α

To understand the underlying mechanism of paroxetine inhibition on M/Lps-induced cytokine expression, we analyzed the mitogen-activated protein kinase (MAPK), NF-κB and STAT3 signaling pathways. Cells were pretreated with paroxetine for 30 min followed by treatment of M/Lps for the indicated time. As shown in the comparisons of 0 min (Fig. 5), paroxetine alone had little impact on the activation of these proteins. The p38, JNK1/2, ERK1/2, p65/NF-κB, and STAT3 were all markedly activated in primary astrocytes upon M/Lps treatments (Fig. 5a). However, the phosphorylation of p38, JNK1/2, ERK1/2, and STAT3 was not suppressed by paroxetine pretreatment except that of p65/NF-κB (Fig. 5b–f). The inhibition rate for p-p65 was 33.7, 40.2, 49.4, and 53.6%, respectively, at 15, 30, 60, and 120 min [Fig. 5e; M/Lps*time interaction: *F*_(4,30)_ = 3.08, *p* = 0.031; M/Lps + paroxetine vs M/Lps: *p* = 0.001 (15 min), *p* = 0.004 (30 min), *p* < 0.001 (60 min), *p* = 0.001 (120 min)]. Immunofluorescence further showed that M/Lps stimulated nuclear translocation of p65 and this translocation was blocked by paroxetine pretreatment [Fig. 6; time*paroxetine interaction: *F*_(1,8)_ = 12.30, *p* = 0.008; M/Lps + paroxetine vs M/Lps: *p* = 0.012 (30 min)]. Pretreatment of PDTC (a specific inhibitor of NF-κB pathway; Fig. 7a; M/Lps*PDTC interaction: *F*_(1,8)_ = 27.24, *p* = 0.001; M/Lps + PDTC vs M/Lps: *p* < 0.001) reduced the M/Lps-induced elevation of TNF-α and IL-1β mRNA expression by 18.0% and 39.0%, respectively, in the astrocytes (Fig. 7b; TNF-α: *F*_(1,8)_ = 22.14, *p* = 0.002; M/Lps + PDTC vs M/Lps: *p* < 0.001; IL-1β: *F*_(1,8)_ = 18.73, *p* = 0.003; M/Lps + PDTC vs M/Lps: *p* < 0.001).

**Fig. 8:**
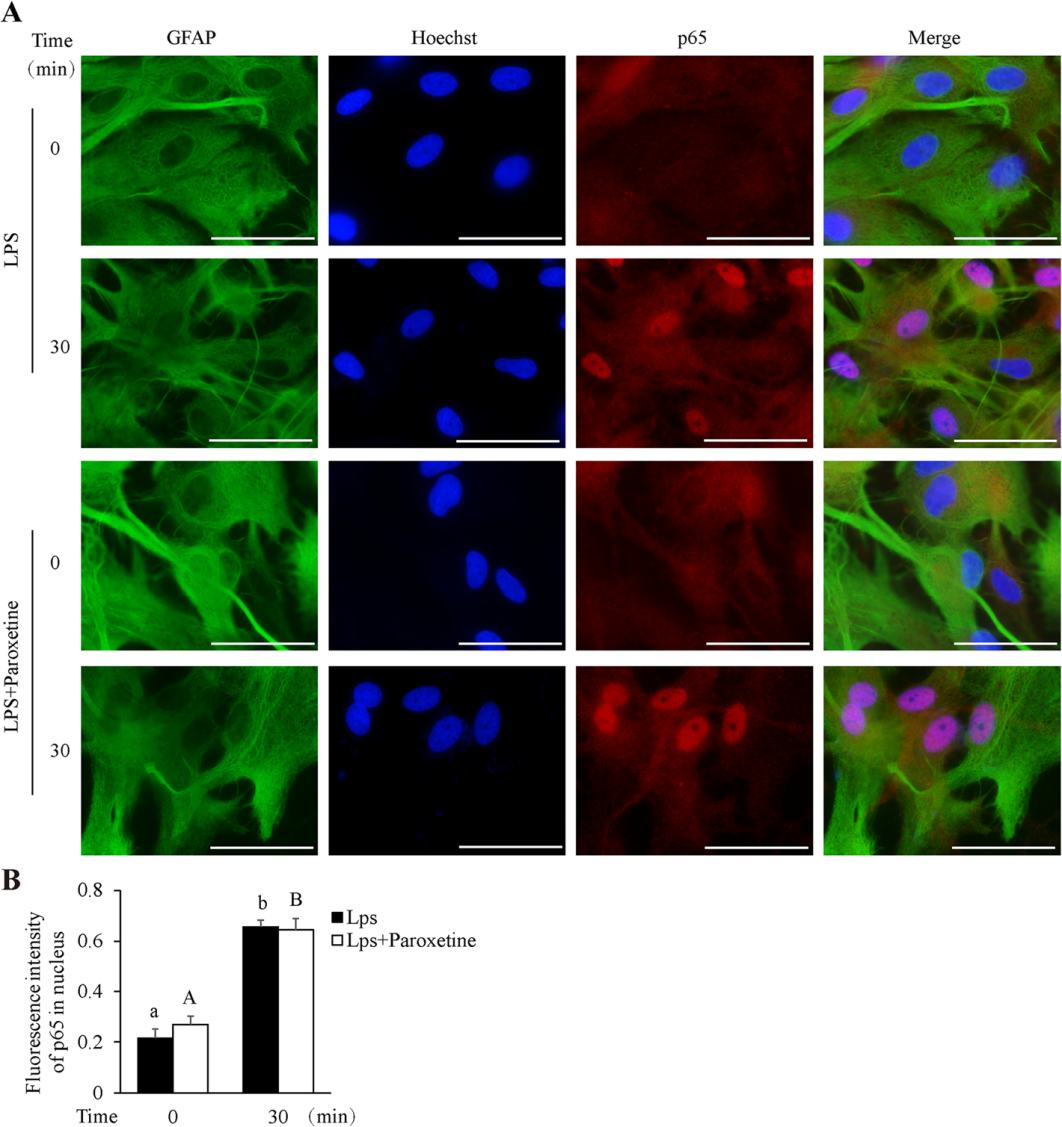
Effect of paroxetine on LPS-induced p65/NF-κB nuclear translocation in primary astrocytes. Cells were pretreated with or without 10 μM of paroxetine for 30 min followed by stimulation with LPS for 30 min. **a** Representative images of p65 nuclear translocation. Green, GFAP; red, p65; blue, nuclei; bar size, 200 μm. **b** Fluorescence intensity of nuclear p65 was determined by quantifying 15 cells in multiple random fields, followed by normalization to their respective nuclei staining. Data are expressed as means ± SE from three independent experiments. Statistical comparisons were performed using two-way ANOVA. Different letters indicate *p* < 0.05. GFAP, glial fibrillary acidic protein; LPS, lipopolysaccharide

We additionally examined the effect of paroxetine on p65/NF-κB activation in the context of LPS stimulation. Results showed that LPS induced little p65 phosphorylation (data not shown) but stimulated apparent p65 nuclear translocation (*p* < 0.05). However, in concord with its effect on cytokine production, paroxetine did not blunt this translocation (Fig. 8).

### A/MLps with paroxetine has little impact on SH-SY5Y cell viability

**Fig. 9:**
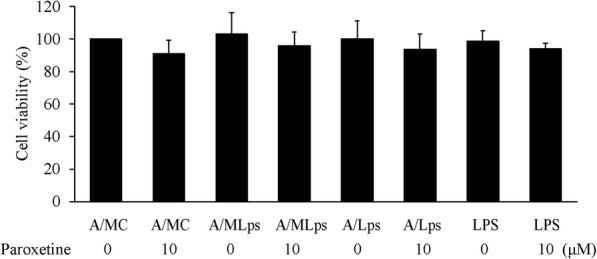
Effect of astrocyte conditioned media on SH-SY5Y cell survival. SH-SY5Y cells were incubated with A/MC, A/MLps, A/Lps, or LPS for 24 h. Cell viability was expressed as percentage of the A/MC alone-treated group, which was set as 100%. Values are means ± SE, *n* = 3. Statistical comparisons were performed using two-way ANOVA. A/Lps, conditioned medium of astrocytes cultured with LPS; A/MC, conditioned medium of astrocytes cultured with M/C; A/MLps, conditioned medium of astrocytes cultured with M/Lps

To investigate whether neuronal survival was affected by the reactive-microglia-mediated activation of astrocytes, LPS-activated astrocytes, and LPS per se, we cultured SH-SY5Y cells, a neuroblastoma cell line commonly used for PD study, with A/MLps, A/Lps, and LPS. Results showed that A/MLps, A/Lps, and LPS regardless of paroxetine pretreatment resulted in little impact on the survival of SH-SY5Y cells (Fig. 9).

**Fig. 10:**
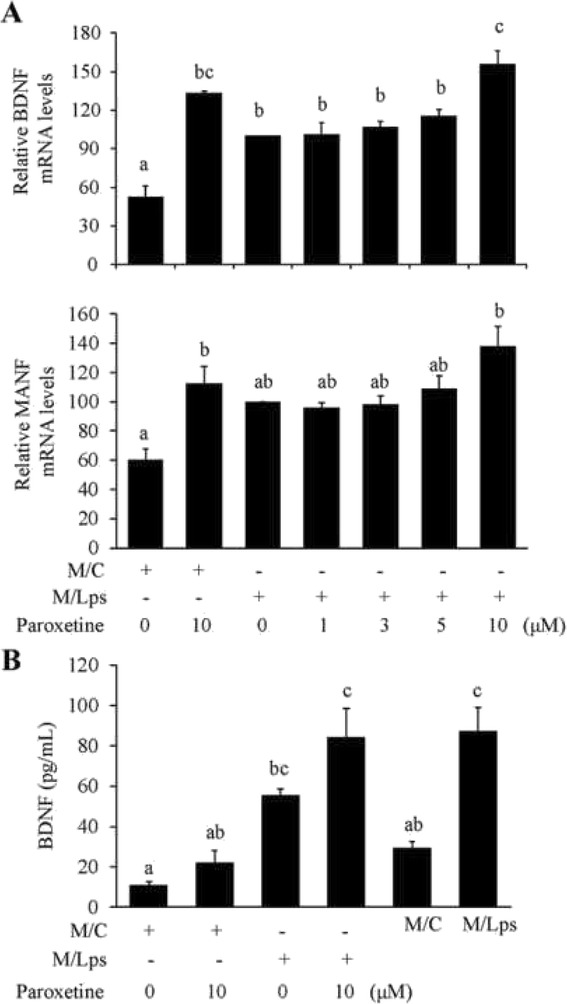
Effect of paroxetine on neurotrophic responses of astrocytes. Cells were pretreated with paroxetine at different concentrations for 30 min followed by incubation with microglia conditioned media for 24 h. **a** Quantitative PCR analyses of BDNF and MANF expression. The mRNA levels were expressed relative to the one incubated with M/Lps alone, which was set as 100. **b** Concentrations of BDNF in culture media. Values are means ± SE, *n* = 3 for **a**, *n* = 4 for **b**. Statistical comparisons were performed using one-way ANOVA, followed by Tukey’s post hoc test. Different letters indicate *p* < 0.05. BDNF, brain-derived neurotrophic factor; MANF, mesencephalic astrocyte-derived neurotrophic factor; M/C, microglia conditioned medium without LPS stimulation; M/Lps, microglia conditioned medium pre-activated with LPS

We further investigated whether potential neurotrophic support of the astrocytes played a role amidst. The mRNA expression of neurotrophic factors, in particular BDNF, was increased in astrocytes following the incubation with M/Lps [Fig. 10a; BDNF: *F*_(6,14)_ = 16.41, *p* < 0.001; post hoc, *p* = 0.012 (M/C vs M/Lps; up by 89%); MANF: *F*_(6,14)_ = 5.06, *p* = 0.006]. In striking contrast to the results in cytokines, paroxetine did not suppress the M/Lps-induced expression of these neurotrophic factors. Instead, paroxetine at 10 μM led to higher mRNA expression of BDNF and MANF in astrocytes [BDNF: post hoc, *p* < 0.001 (M/C vs M/C + paroxetine 10 μM; up by 152%), *p* = 0.003 (M/Lps vs M/Lps + paroxetine 10 μM; up by 56%); MANF: *p* = 0.037 (M/C vs M/C + paroxetine 10 μM; up by 88%)]. Similarly, paroxetine at 10 μM resulted in a trend of elevation in medium BDNF levels compared with their respective controls (Fig. 10b). BDNF concentration was significantly increased in media of the astrocytes incubated with M/Lps compared to the M/C incubation (*F*_(5,18)_ = 15.61, *p* < 0.001; post hoc, *p* = 0.003). Interestingly, compared to the baseline levels in the conditioned media, medium BNDF levels appeared to be reduced upon incubation with the astrocytes (Fig. 10b; the first vs fifth column, the third vs sixth column).

## Discussion

Neuroinflammation is involved in the pathophysiology of neurodegenerative diseases such as PD and Alzheimer’s disease [1–3]. Targeting microglia- and astrocyte-mediated immune responses is therapeutically promising against these disorders [42]. Besides being a drug against depression, paroxetine is shown to inhibit MPTP-induced loss of nigrostriatal dopaminergic neurons and neuroinflammatory responses, and to suppress LPS-induced microglia activation [28, 36, 43]. Results of the current study in primary astrocytes suggest that (1) paroxetine inhibits reactive microglia-mediated, but not LPS-induced, astrocytic inflammatory responses; (2) LPS-induced microglia activation dominates over its subsequent activation of astrocytes; (3) paroxetine-mediated astrocytic responses during LPS insult has a little actual impact on neuronal survival.

Astrocytes are often activated following activation of microglia, which may stimulate astrocytes in a paracrine pathway [44]. Incubation of astrocytes with microglia conditioned medium indicates a paracrine regulation of microglia on astrocytic responses. Indeed, reactive microglia stimulate mRNA expression of both cytokines such as TNF-α and IL-1β and neurotrophic factors such as BDNF and MANF in astrocytes. However, compared to the reactive microglia per se, this paracrine activation of astrocytes may have a rather small effect on the surroundings as suggested by the light extracellular release of effectors in media. It is not surprising given that microglia are presumably more potent than astrocytes leading to neuroinflammation [4, 5]. In consistency, the induction of iNOS by LPS in astrocytes is considerably less than that in microglia [36], which may explain why the NO production is not significantly elevated in astrocytes. Sheng et al. also showed that NO production was not detectable in either immortalized rat astrocytes or primary astrocytes upon treatment of LPS at 100 ng/mL [45].

However, while reactive microglia indeed release neurotrophic factors [46, 47], we initially thought that astrocytes should have been more potent secreting neurotrophic factors upon being activated. Unexpectedly, there is no drastic increase of BDNF in the astrocyte media compared to its baseline levels of microglia conditioned media. Instead, astrocytes seem to absorb microglia-released neurotrophic factors as observed. On the other hand, paroxetine indeed upregulates mRNA expression and extracellular release of neurotrophic factors such as BDNF as being previously noted [48].

Saijo et al. reported that microglia-mediated inflammatory responses were magnified by astrocytes leading to more neuronal death [19]. However, as above discussed, this magnification is not displayed in the current study. Both studies used primary astrocytes and BV2 cells for preparing conditioned media. In an effort to interpret the above discrepancy, we observed that their BV2 cells used to make conditioned media were all infected with lentivirus [19]. As manifested in one of their results, LPS-activated conditioned media from this type of cells exhibited significantly higher toxicity to tyrosine hydroxylase positive neurons compared to those prepared from uninfected cells, suggesting that microglia upon lentivirus infection are more reactive and potent generating pro-inflammatory cytokines. Consequently, paracrine factors in their microglia conditioned media are presumably of much higher concentrations, then leading to more drastic astrocytic responses than in the current study. BV2 cell is considered as a valid substitute for primary microglia in complex cell-cell interaction studies [49]. Nonetheless, the obtained results should be confirmed using conditioned medium derived from primary microglia cultures.

Paroxetine prevents LPS-induced inflammatory responses in microglia [36], but not in astrocytes. Toll-like receptor distribution varies considerably between microglia and astrocytes [50]. For instance, TLR4, the major LPS receptor, is expressed extensively in microglia, but this receptor and its downstream signaling component MYD88 are deficient in rodent astrocytes [51, 52]. Thus, the signaling pathways in response to LPS much differ in these two types of immune cells. Paroxetine markedly suppresses the reactive microglia-induced activation of p65/NF-κB in astrocytes including both phosphorylation and nuclear translocation. Interestingly, the nuclear translocation of p65 induced by a direct LPS treatment was not blunted by paroxetine. These results suggest that the two kinds of stimuli, LPS and cytomix from conditioned media, have different intermediate effectors in astrocytes. Completely blocking NF-κB pathway by PDTC results in less inhibition in cytokine expression compared to those by paroxetine (18.0% vs 42.2%, and 39.0% vs. 61.8%, respectively, for the reductions in TNF-α and IL-1β expression). Thus, additional pathways should be involved in the paroxetine suppression of astrocytic inflammatory responses. STAT3 and MAPKs including p38, JNK, and ERK are considered to be key regulators in astrocytes in response to neuroinflammation besides NF-κB [53]. However, it turns out that both MAPKs and STAT3 are not the answer. Paroxetine is also reported to prevent the downregulation of L-Glu transporters in astrocytes by inhibiting L-Glu release from reactive microglia, which otherwise causes excitotoxicity [54, 55]. In addition, paroxetine antagonizes P2X_4_ receptor, resulting in inhibition of ATP-induced calcium influx in 1321 N1 astrocytic cell line [56]. Thus, further investigation is needed to fully understand signaling participated in this regulation by paroxetine.

We have previously reported that incubation with LPS-stimulated microglia conditioned media leads to a significant death of SH-SY5Y cells by about 15%, where for example the medium TNF-α level is at approximately 8500 pg/mL [36]. No significant impact on SH-SY5Y cell viability is observed when incubated with the reactive microglia- or LPS-stimulated astrocyte conditioned media, where the level of TNF-α is roughly only at 1100 and 100 pg/mL, respectively, as a comparison. Minor amounts of cytokines and neurotrophic factors are derived from astrocytes as above mentioned. Paroxetine thus renders little impact on the neuronal survival under this condition.

## Conclusions

The current study demonstrates that paroxetine ameliorates the reactive microglia-mediated inflammatory responses in astrocytes partially via inhibition of NF-κB pathway, but has no impact on LPS-stimulated astrocyte activation. Paroxetine also stimulates neurotrophic support of astrocytes. While the effect of paroxetine on this secondary astrocytic response appears minor compared to its effect on the innate immune responses of microglia, paroxetine can indeed alleviate neuroinflammation by suppressing microglia activation [36] and its paracrine inflammatory activation of astrocytes. The microglia and astrocyte results combined may implicate a therapeutic potential of paroxetine against neuroinflammation-associated neurological disorders such as PD.

## Supplementary information

**Additional file 1:.** Figure S1. Full blots for the Figs. 3a, 6a and 7a.

## Data Availability

All data supporting the conclusions of this study are included in the article.

## Abbreviations

A/Lps: Conditioned medium of astrocytes cultured with LPS
A/MC: Conditioned medium of astrocytes cultured with M/C
A/MLps: Conditioned medium of astrocytes cultured with M/Lps
BDNF: Brain-derived neurotrophic factor
ERK: Extracellular signal-regulated kinase
GFAP: Glial fibrillary acidic protein
IL-1β: Interleukin 1β
iNOS: Inducible nitric oxide synthase
JNK: c-jun N-terminal kinase
LPS: Lipopolysaccharide
M/C: Microglia conditioned medium without LPS stimulation
M/Lps: Microglia conditioned medium pre-activated with LPS
MANF: Mesencephalic astrocyte-derived neurotrophic factor
MAPK: Mitogen-activated protein kinase
NF-κB: Nuclear factor κB
NO: Nitric oxide
PD: Parkinson’s disease
PDTC: Pyrrolidine dithiocarbamic acid
STAT3: Signal transducer and activator of transcription 3
TLR: Toll-like receptor
TNF-α: Tumor necrosis factor α
