# Supplementary figures and images for: Paroxetine suppresses reactive microglia-mediated but not lipopolysaccharide-induced inflammatory responses in primary astrocytes
Source: J Neuroinflammation. 2020 Feb 5;17:50. doi: 10.1186/s12974-020-1712-0 (PMC7003432; doi:10.1186/s12974-020-1712-0)

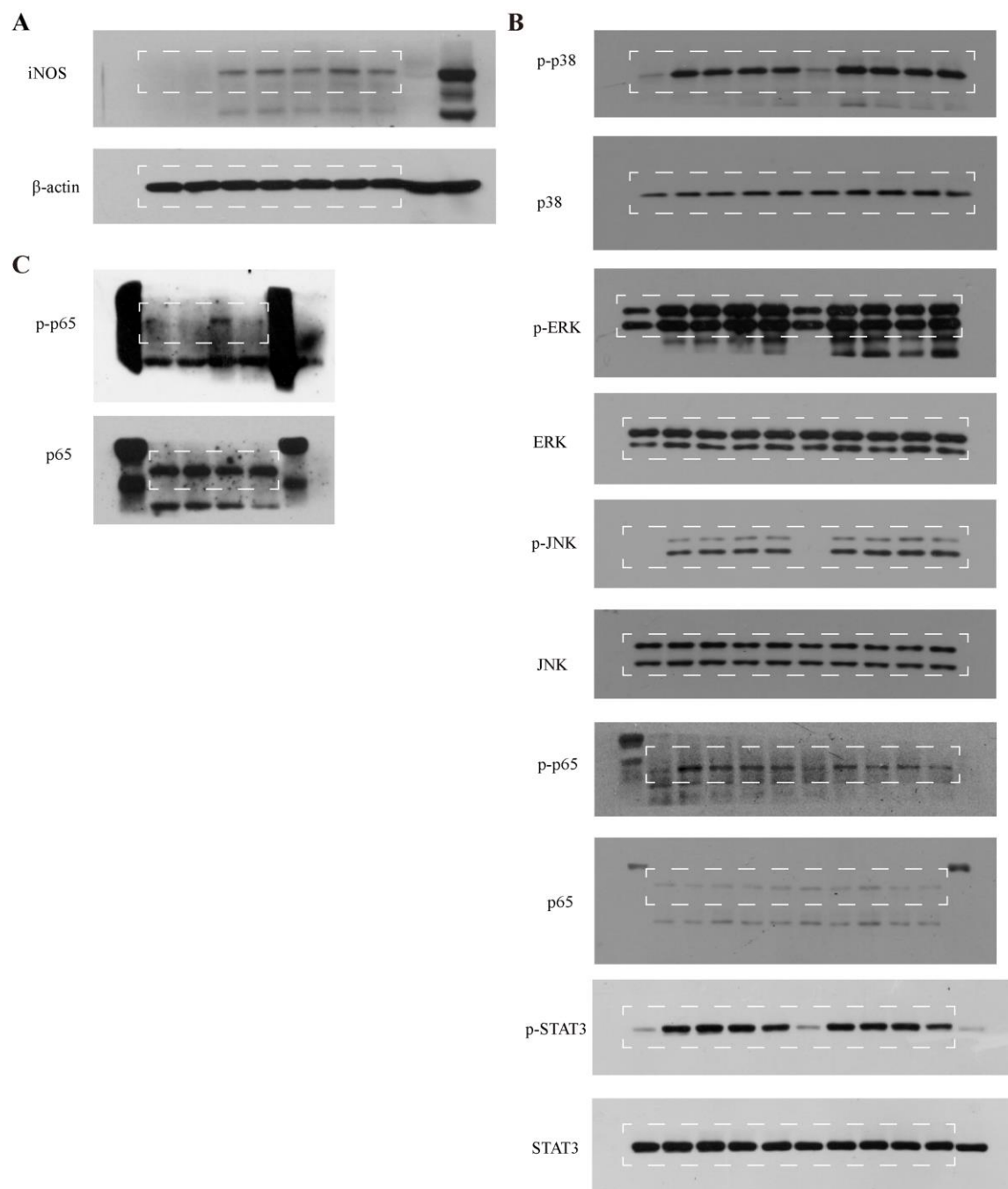

**Figure S1.** Full blots for the figures 3A (A), 6A (B) and 7A (C).

Supplement: Supplementary file 1 — Additional file 1:. Figure S1. Full blots for the Figs. 3a, 6a and 7a. [file 12974_2020_1712_MOESM1_ESM.pdf]
